# Associations between life course longitudinal growth and hip shapes at ages 60–64 years: evidence from the MRC National Survey of Health and Development

**DOI:** 10.1136/rmdopen-2023-003816

**Published:** 2024-04-10

**Authors:** Katherine Ann Staines, Fiona R Saunders, Alex Ireland, Richard M Aspden, Jennifer S Gregory, Rebecca J Hardy, Rachel Cooper

**Affiliations:** 1 Centre for Lifelong Health, School of Applied Sciences, University of Brighton, Brighton, UK; 2 Centre for Arthritis & Musculoskeletal Health, School of Medicine, Medical Sciences & Nutrition, University of Aberdeen, Institute of Medical Sciences, Aberdeen, UK; 3 Department of Life Sciences, Musculoskeletal Science and Sports Medicine Research Centre, Manchester Metropolitan University, Manchester, UK; 4 School of Sport, Exercise and Health Sciences, Loughborough University, Loughborough, UK; 5 AGE Research Group, Translational and Clinical Research Institute, Faculty of Medical Sciences, Newcastle University, Newcastle upon Tyne, UK; 6 NIHR Newcastle Biomedical Research Centre, Newcastle upon Tyne NHS Foundation Trust, Cumbria, Northumberland, Tyne and Wear NHS Foundation Trust and Newcastle University, Newcastle, UK

**Keywords:** Osteoarthritis, Epidemiology, Chondrocytes

## Abstract

**Objective:**

We sought to examine associations between height gain across childhood and adolescence with hip shape in individuals aged 60–64 years from the Medical Research Council National Survey of Health and Development, a nationally representative British birth cohort.

**Methods:**

Height was measured at ages 2, 4, 6, 7, 11 and 15 years, and self-reported at age 20 years. 10 modes of variation in hip shape (HM1–10), described by statistical shape models, were previously ascertained from DXA images taken at ages 60–64 years. Associations between (1) height at each age; (2) Super-Imposition by Translation And Rotation (SITAR) growth curve variables of height size, tempo and velocity; and (3) height gain during specific periods of childhood and adolescence, and HM1–10 were tested.

**Results:**

Faster growth velocity was associated with a wider, flatter femoral head and neck, as described by positive scores for HM6 (regression coefficient 0.014; 95% CI 0.08 to 0.019; p<0.001) and HM7 (regression coefficient 0.07; 95% CI 0.002 to 0.013; p=0.009), and negative scores for HM10 (regression coefficient −0.006; 95% CI −0.011 to 0.00, p=0.04) and HM2 (males only, regression coefficient −0.017; 95% CI −0.026 to −0.09; p<0.001). Similar associations were observed with greater height size and later height tempo. Examination of height gains during specific periods of childhood and adolescence identified those during the adolescence period as being most consistently associated.

**Conclusion:**

Our analyses suggest that individual growth patterns, particularly in the adolescent period, are associated with modest variations in hip shape at 60–64 years, which are consistent with features seen in osteoarthritis.

**WHAT IS ALREADY KNOWN ON THIS TOPIC**

- Joint shape is closely related to osteoarthritis predisposition.
- Emerging evidence has suggested an association between endochondral ossification and longitudinal bone, and osteoarthritis predisposition.

**WHAT THIS STUDY ADDS**

- Individual growth patterns, particularly in the adolescent period, are associated with hip shapes which have features consistent with those seen in osteoarthritis.

**HOW THIS STUDY MIGHT AFFECT RESEARCH, PRACTICE OR POLICY**

- This research suggests that individual growth trajectories may predict osteoarthritis risk and therefore allow prediction of health and well-being needs throughout the life course.

## Introduction

Osteoarthritis, characterised by articular cartilage loss, subchondral bone thickening and osteophyte formation, is a global healthcare burden affecting about 528 million people worldwide in 2019.^1^ There are currently no disease-modifying treatments available, and once disease has advanced, total joint replacement is the only option. Therefore, a better understanding of the mechanisms underpinning osteoarthritis, and in particular identification of predictors of disease risk, could help inform strategies for prevention and treatment.

Joint shape is known to be closely related to osteoarthritis predisposition. Indeed, developmental dysplasia of the hip is a known risk factor for both human and animal osteoarthritis.^2^ Similarly, we and others have shown in numerous studies, using statistical shape modelling (SSM), a sophisticated technique that enables identification of subtle shape variations, that hip shape is associated with radiographic osteoarthritis.^3–5^ There is also evidence of an association between cam morphology (bulging of the lateral femoral head) and hip osteoarthritis.^6^ Cam morphology develops around the time of epiphysial growth plate closure, suggestive of correlation between endochondral ossification, subsequent cam morphology and osteoarthritis development.^7–10^ Recent genetic studies have further indicated associations between hip shape and the process of endochondral ossification.^11–13^ Concurrent with this, a study from offspring in the Avon Longitudinal Study of Parents and Children (ALSPAC) found height tempo (corresponding to pubertal timing) to be strongly associated with the hip shape modes which have previously been shown to be related to an individual’s risk of developing hip osteoarthritis in ageing.^14^ Moreover, we have examined associations between lifetime linear growth trajectories using the Super-Imposition by Translation And Rotation (SITAR) variables of height size, tempo and velocity, and knee osteoarthritis in the Medical Research Council (MRC) National Survey of Health and Development (NSHD). We found that increased height in childhood was associated, although modestly, with lower odds of knee osteoarthritis at age 53 years, as was adult achieved height.^13^ Adult achieved height has also been shown to correlate with subtle variations in hip shape in this cohort.^4^ Similarly, age at onset of walking is associated with hip shape in early old age in this cohort, and these associations were not explained by adjustment for lean mass, suggesting that early life skeletal loading may play a key role in the underlying mechanism.^15^ Whether linear growth and in particular, its timing and velocity, during childhood and adolescence are associated with hip shape is unknown.

This has therefore led us to hypothesise that life course longitudinal growth may be associated with variations in hip shape relevant to the development of osteoarthritis, which has important implications for musculoskeletal health in aged individuals. Here, we interrogated the MRC NSHD to examine the relationships between height gain across childhood and adolescence with hip shape in individuals aged 60–64 years. Our aims were to test associations of: (1) achieved height at specific ages during childhood and adolescence; (2) SITAR parameters representing differences in height (*height size*), pubertal timing (*height tempo*) and rate of growth (*height velocity*); (3) height gain during specific life periods, with hip shapes characterised using SSM of DXA images taken at age 60–64 years.

## Materials and methods

### Study sample

The MRC NSHD is a birth cohort study, which includes a nationally representative sample of 2815 men and 2547 women born in England, Scotland and Wales during 1 week in March 1946 (online supplemental figure 1).^16 17^ The cohort has been followed prospectively across life with outcome data for these analyses drawn from a data collection between 2006 and 2010. Between these dates, eligible participants known to be alive and living in England, Scotland and Wales were invited for an assessment at one of six clinical research facilities. Of 2856 individuals invited, 1690 attended a clinical research facility and 539 received a home visit from a research nurse (online supplemental figure 1).

10.1136/rmdopen-2023-003816.supp1 Supplementary data

### Statistical shape modelling

During their visit to a clinical research facility at age 60–64 years, images of the participant’s total body and left hip (except in 63 cases where contraindication of a prosthesis meant that the right hip was scanned) were obtained using a QDR 4500 Discovery DXA scanner (Hologic, Bedford, Massachusetts, USA) (online supplemental figure 1). All hip scans were performed with the feet placed at 15° of internal rotation. In five centres, scanners had rotating C‐arms allowing participants to lie supine for all scans; one centre used a scanner with a fixed C‐arm. Of the 1690 participants who attended a clinical research facility, 1636 had a hip DXA scan (online supplemental figure 1). Three images were excluded because of extreme internal rotation of the femur, evidenced by femoral neck foreshortening, leaving 1633 images to build the hip SSM, as previously described.^4^ Briefly, a template consisting of 68 points describing the major anatomical landmarks was applied to each image and the points manually corrected. The statistical shape model was built by first transforming the points using the Procrustes transform and then principal component analysis was performed to derive the orthogonal modes of variation. Hip mode (HM) scores were normalised to a mean of 0 and SD of 1, each accounted for >2% hip shape variation. In total, these 10 modes accounted for 80.6% of the total variance.

### Height

Height (cm) was measured by nurses using standardised protocols at ages 2, 4, 6, 7, 11 and 15 years, and self-reported at age 20 years. Individual patterns of height growth during puberty were estimated using the SITAR model of growth curve analysis, as previously described by Cole *et al*.^18 19^ The SITAR model uses a natural cubic spline to model the growth curve of height against age. It estimates the mean growth curve and three individual specific parameters which make all individual growth curves as similar as possible. The individual parameters, expressed relative to the mean growth curve, are *height size* (representing differences in height), *height tempo* (representing differences in the age at peak height velocity and thus differences in pubertal timing) and *height velocity* (representing differences in rate of growth).

### Covariates

Factors that have previously been shown to be associated with both the main explanatory factors and with hip shape and so may potentially confound the main associations of interest were selected a priori based on previous findings in the literature.^13 15 20–22^ These were early life factors–birth weight, father’s occupational class in childhood (categorised as non-manual vs manual) and sporting ability at 13 years (categorised as above average, average or below average by their teachers completing a questionnaire rating their ability in school sports compared with their peers).^23 24^ In our final models, we further adjusted for weight. Weight was measured by nurses using standardised protocols at ages 2, 4, 6, 7, 11 and 15 years, and self-reported at age 20 years. As above, individual patterns of weight growth during puberty were estimated using the SITAR model of growth curve analysis.^18 19^ The SITAR model estimates: *weight size* (an individual’s mean weight compared with the average), *weight tempo* (an individual’s age at peak weight velocity compared with the average) and *weight velocity* (an individual’s weight growth rate compared with the average), each expressed relative to the mean curve.

### Statistical analysis

We used linear regression models to test associations between: (1) height at each age, (2) SITAR height variables and (3) conditional changes in height during specific periods of early life (early childhood: 2–4 years; late childhood: 4–7 years; childhood to adolescence: 7–15 years; adolescence to young adulthood: 15–20 years) and each hip shape mode.

In models to address (3), we generated conditional changes in height by regressing each height measure on the earlier height measure for each sex and calculating the residuals.^25^ The residuals were standardised (to have mean 0 and SD of 1) to ensure their comparability and these were included as the main independent variables.

In initial models, we formally tested for interactions between sex and each main independent variable and where no evidence of interaction was found based on statistical significance (p<0.05), models were fitted with men and women combined and adjusted for sex. We also tested for deviations from linearity by including quadratic terms in all growth variables. In each set of models, we first adjusted for sex (where there was no evidence of interaction), before then also adjusting for early life factors (birth weight+sporting ability at 13 years+father’s occupational class in childhood). In our final model, we adjusted SITAR height for SITAR weight variables, and conditional height gain for conditional weight gain to assess the contribution of weight during growth. To maximise sample size when examining height at each age, each set of models was run on the sample with valid data for the height at that age, the HM and the covariates. For the SITAR analysis, models were run on the sample with valid data for SITAR variables, HM and the covariates (online supplemental figure 1). For the analysis of height gains during specific periods, models were run on the sample with valid data for height at all ages, HM and the covariates to ensure the same N to aid formal comparison of effect sizes across time periods (online supplemental figure 1). All associations are presented in the online supplemental filesonline supplemental files. Data were analysed using Stata statistical software (V.SE 14.2).

## Results

Characteristics of the participants included in this study are detailed in table 1. A total of 770 men and 842 women had complete data on the SITAR parameters of height and the hip shape modes. In this sample, women were on average shorter than men and had a lower mean birth weight (table 1).

**Table 1:** Characteristics of the sample from the MRC National Survey of Health and Development with complete data on the SITAR height parameters and hip modes (maximum n=769; N varies due to missing data)

		Men			Women		
		N	Mean	SD	N	Mean	SD
Height (cm) at age (years):							
2		638	86.0	5.0	684	85.0	4.4
4		690	103.7	4.8	752	103.5	4.9
6		658	114.8	5.0	716	114.4	5.2
7		673	120.7	5.2	743	120.4	5.4
11		659	141.2	6.6	722	142.0	6.9
15		616	162.6	8.9	666	159.4	6.0
20		639	177.3	6.5	728	163.1	6.1
Birth weight (kg)		769	3.5	0.5	838	3.4	0.5
Weight (kg) at age (years):							
2		659	13.2	1.4	714	12.7	1.5
4		713	17.6	2.1	769	17.2	2.1
6		663	21.0	2.5	724	20.5	2.6
7		657	23.1	2.8	718	22.8	3.1
11		659	34.4	5.8	716	35.3	6.8
15		618	51.2	9.2	665	52.3	8.1
20		642	70.4	8.7	723	57.8	8.2
		**N**	**%**		**N**	**%**	
Sporting ability at 13 years	Above average	141	20.9		131	18.0	
	Average	417	61.8		527	72.5	
	Below average	117	17.3		69	9.5	
Father’s occupational class in childhood	Manual	360	48.7		400	49.7	
	Non-manual	379	51.3		405	50.3	

MRC, Medical Research Council; SITAR, Super-Imposition by Translation And Rotation.

Overall, there was evidence that variations in the patterns of pubertal growth and in height at different ages across childhood and adolescence were associated with HM scores at age 60–64 years, with most associations maintained when adjusted for life factors and weight (tables 1–3 and online supplemental tables 2–4). However, not all associations were found to be equally strong and there was some evidence of deviations from linearity when quadratic terms were included in our models (online supplemental table 1). When plots of the associations between height and HM were examined in those cases where the quadratic term was statistically significant, this suggested that any deviations from linearity were minor and due to extreme values (online supplemental figure 2).

**Figure 1:**
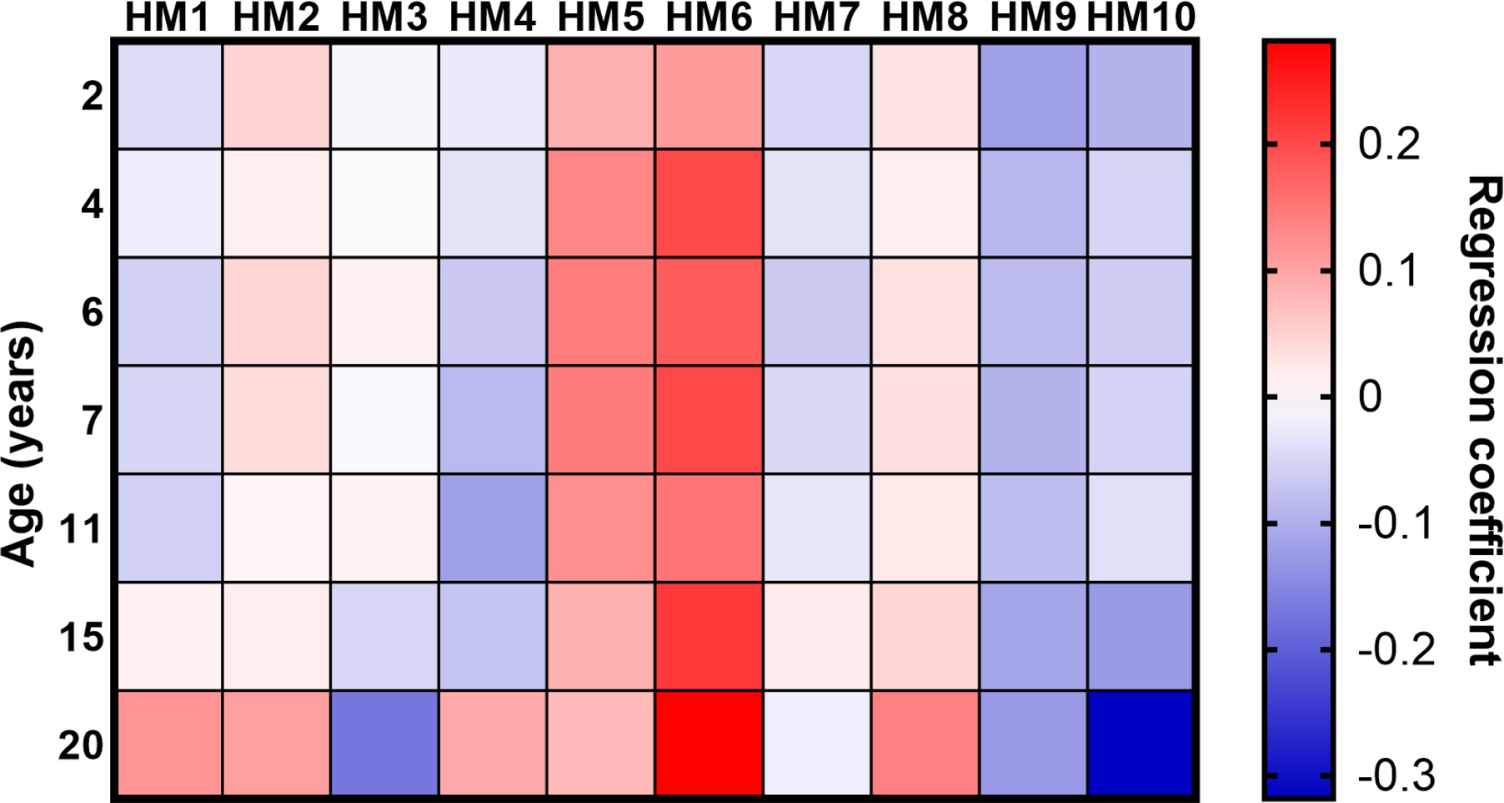
Heat map of the associations between height at ages 2–20 years and each hip shape mode (HM1–HM10). Drawn from the regression coefficients for associations between height assessed at ages 2, 4, 6, 7, 11, 15 and 20 and each hip shape mode score.

In models adjusted for sex and early life factors, height at all ages was associated with positive HM5 and HM6, and negative HM9 scores (figure 1, online supplemental table 2, online supplemental figure 3). In models of HM2, there was evidence of interactions between sex and height at all ages from 6 years onwards (online supplemental table 2). Associations were negative in men and positive in women, with evidence that it was the positive associations in women that were stronger (online supplemental table 2). In all other HMs, the strongest associations varied by age of height assessment, and these tended to be for measures of height in adolescence rather than childhood (figure 1 and online supplemental table 2).

We next examined the SITAR parameters of *height size*, *tempo* and *velocity* with each HM (table 2 and online supplemental figure 4). When adjusted for sex and early life factors, greater *height size* was associated with negative mean scores for HM1, HM9 and HM10, and positive scores for HM6 and HM8. In models of HM2, there was evidence of interactions between sex and both *height size* (p=0.007) and *height velocity* (p=0.007). In both sexes, associations were negative but there was evidence that the associations were stronger in men than women. Greater *height velocity* was associated with positive mean scores for HM6 and HM7, and negative mean scores for HM10. Later *height tempo* was associated with negative mean scores for HM2; however, there were no associations with any of the other HMs.

**Table 2:** Associations between each parameter of the SITAR model of growth curve analysis (per 1 unit change in height size (cm), tempo (years) and velocity (%)) and HM1–10

Mode	SITAR	RC	95% CI		P value
HM1	Size	−0.012	−0.023	−0.002	**0.03**
	Tempo	0.002	−0.008	0.012	0.7
	Velocity	−0.004	−0.010	0.001	0.1
HM2	Size (M)	−0.041	−0.058	−0.025	**<0.001**
	Size (F)	−0.013	−0.028	0.001	0.07
	Tempo	−0.015	−0.025	−0.005	**0.003**
	Velocity (M)	−0.017	−0.026	−0.009	**<0.001**
	Velocity (F)	−0.003	−0.010	0.003	0.4
HM3	Size	0.004	−0.007	−0.015	0.5
	Tempo	0.001	−0.009	0.010	0.9
	Velocity	0.002	−0.003	0.008	0.4
HM4	Size	−0.003	−0.014	0.007	0.5
	Tempo	−0.003	−0.012	0.007	0.6
	Velocity	−0.004	−0.010	0.001	0.1
HM5	Size	0.006	−0.005	0.017	0.3
	Tempo	0.007	−0.003	0.017	0.2
	Velocity	−0.002	−0.007	0.004	0.5
HM6	Size	0.039	0.028	0.049	**<0.001**
	Tempo	0.005	−0.005	0.014	0.4
	Velocity	0.014	0.008	0.019	**<0.001**
HM7	Size	0.009	−0.003	0.020	0.1
	Tempo	0.004	−0.006	0.014	0.5
	Velocity	0.007	0.002	0.013	**0.009**
HM8	Size	0.017	0.006	0.028	**0.002**
	Tempo	−0.002	−0.013	0.008	0.6
	Velocity	0.004	−0.002	0.009	0.2
HM9	Size	−0.012	−0.023	−0.001	**0.04**
	Tempo	0.008	−0.002	0.018	0.1
	Velocity	−0.003	−0.009	0.002	0.3
HM10	Size	−0.015	−0.025	−0.005	**0.005**
	Tempo	0.002	−0.008	0.011	0.8
	Velocity	−0.006	−0.011	0.000	**0.04**

All models were run on the sample with valid data for each hip shape mode, each SITAR variable and the confounders. Data presented are from linear regression models adjusted for sex (unless sex interactions were observed), birth weight, sporting ability and father’s occupational class in childhood, as well as SITAR weight. n=1380 (667 male and 713 female). Data from models 1 and 2 are presented in online supplemental table 3. Significant p values are highlighted in bold.

HM, hip mode; RC, regression coefficient; SITAR, Super-Imposition by Translation And Rotation.

When examining height gains between specific growth periods, no patterns were consistently found. Where there was any suggestion of an association, this was typically found in relation to changes in height between 7 and 15 years, suggesting these conditional height gains in adolescence rather than childhood may be more important in defining hip shape (HM2 (p<0.001), HM5 (p=0.03), HM6 (p=0.05) and HM7 (male only: p<0.001); table 3). Subsequent formal comparisons of height gain between 7 and 15 years compared with other periods showed evidence that the association at 7–15 years was different from the association in all other periods with regard to HM5 and HM7 (p<0.05), with the size of the effect estimates suggesting a stronger association at 7–15 years. However, with regard to HM6, the size of the effect estimates suggested a stronger association at 2–4 years than at 7–15 years (p=0.03).

**Table 3:** Associations of conditional height gain (per SD) during different periods of growth (early childhood: 2–4 years; late childhood: 4–7 years; childhood to adolescence: 7–15 years; adolescence to young adulthood: 15–20 years) with each hip shape mode

Mode	Conditional change (years)	RC	95% CI		P value
HM1	2–4	0.021	−0.060	0.102	0.6
	4–7	−0.051	−0.134	0.032	0.2
	7–15	−0.030	−0.115	0.055	0.5
	15–20	−0.016	−0.098	0.067	0.7
HM2	2–4	−0.046	−0.128	0.036	0.3
	4–7 (M)	−0.009	−0.127	0.109	0.9
	4–7 (F)	0.058	−0.062	0.179	0.3
	7–15	−0.115	−0.201	−0.030	**<0.001**
	15–20	−0.129	−0.212	−0.046	**0.002**
HM3	2–4	0.010	−0.068	0.089	0.8
	4–7	−0.051	−0.131	0.029	0.2
	7–15	−0.006	−0.009	0.077	0.9
	15–20	0.004	−0.076	0.084	0.9
HM4	2–4	0.007	−0.074	0.089	0.9
	4–7	−0.052	−0.134	0.030	0.2
	7–15 (M)	−0.028	−0.176	0.120	0.7
	7–15 (F)	−0.011	−0.114	0.091	0.8
	15–20	−0.017	−0.100	0.065	0.7
HM5	2–4	0.062	−0.021	0.145	0.1
	4–7	−0.022	−0.106	0.062	0.6
	7–15	−0.094	−0.181	−0.075	**0.03**
	15–20	0.045	−0.039	0.130	0.3
HM6	2–4	0.138	0.059	0.217	**0.001**
	4–7	0.049	−0.033	0.131	0.2
	7–15	0.086	0.002	0.170	**0.05**
	15–20	0.071	−0.010	0.152	0.09
HM7	2–4	−0.009	−0.073	0.092	0.8
	4–7	−0.019	−0.104	0.065	0.7
	7–15 (M)	0.249	0.116	0.382	**<0.001**
	7–15 (F)	0.044	−0.075	0.163	0.5
	15–20	0.028	−0.056	0.113	0.5
HM8	2–4	0.061	−0.020	0.142	0.1
	4–7	0.016	−0.067	0.098	0.7
	7–15	0.007	−0.079	0.092	0.9
	15–20	0.071	−0.011	0.154	0.09
HM9	2–4	−0.018	−0.101	0.065	0.7
	4–7	−0.035	−0.120	0.050	0.4
	7–15	−0.020	−0.107	0.068	0.7
	15–20	0.077	−0.007	0.162	0.07
HM10	2–4	−0.004	−0.079	0.071	0.9
	4–7	−0.030	−0.106	0.047	0.4
	7–15	−0.061	−0.139	0.018	0.1
	15–20	−0.068	−0.143	0.008	0.08

All models were run on the sample with complete data for each hip shape mode, height at each age and all confounders. Data presented are from linear regression models adjusted for sex (unless sex interactions were observed), birth weight, sporting ability and father’s occupational class in childhood, as well as conditional weight gain. n=648 (319 male and 329 female).

HM, hip mode; RC, regression coefficient.

**Figure 2:**
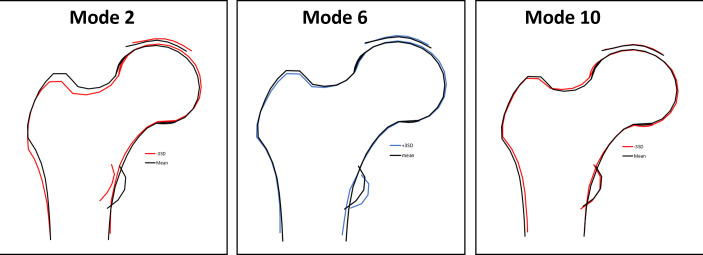
Variations in the modes with the most frequent associations with the SITAR model of growth curve analysis (height size, tempo and velocity) and conditional height gains. Variations in shape for +3 (red) and −3 (blue) SDs in mode score from the mean of 0 for modes 2 and 10 (negative), and 6 (positive). Descriptions of the key features identified by each mode are given in online supplemental table 5. SITAR, Super-Imposition by Translation And Rotation.

When taking the SITAR findings together, in individuals who are taller, have later pubertal timing (later height tempo) or faster growth velocity, the modes with the most frequent associations (HM2, HM6 and HM10) describe features more consistent with those seen in osteoarthritis, with a wider and flatter femoral head and neck, and increased external rotation as indicated by a larger lesser trochanter (figure 2). Consistent with these associations with pubertal timing and faster pubertal growth, the majority of associations with hip shape modes were with conditional height gains in adolescence rather than childhood, and greater gains were also associated with osteoarthritis-like features. The shape variations described by the associations with each of the HM are shown in online supplemental table 5.

## Discussion

In this study, we aimed to examine associations between longitudinal growth during childhood and adolescence and hip shape at age 60–64 years, assessed by SSM from regional DXA scans in the MRC NSHD British birth cohort. Overall, there was evidence that variations in the patterns of pubertal growth and in height at different ages across childhood and adolescence were modestly associated with HM scores at age 60–64 years. Associations were maintained after adjustment for early life factors as well as weight. Examination of height gains during specific periods of childhood and adolescence identified the majority of associations to be in the adolescent period, rather than in childhood.

In both sexes, we observed that faster growth rate (SITAR *height velocity*) was associated with changes in HMs with features more consistent with those seen in osteoarthritis, such as a wider and flatter femoral head and neck, and increased external rotation. Similar associations were observed with greater *height size* and later puberty (SITAR *height tempo*).

These features in the hip shapes are similar to those found to be associated with osteoarthritis development in later life in several other studies.^26^ In summary, in the Rotterdam Study, a cohort of individuals with no signs of radiographic hip osteoarthritis at baseline and using an active shape model, associations between shape changes in the femoral head and neck, and osteoarthritis onset were examined. The study found that significant changes in the shape of the proximal femur occurred within those individuals who developed osteoarthritis.^3^ Similarly, in the Johnston County Osteoarthritis Project, variations with hip shape were associated with prevalent knee radiographic osteoarthritis,^27^ and in a high bone mass cohort, there was strong evidence of an association between cam morphology (bulging of the lateral femoral head) and radiographic hip osteoarthritis, consistent with other studies.^6^ However, there was no evidence of an association between high bone mass and cam morphology, suggestive of distinct pathways by which the risk of osteoarthritis is conferred by these.^6^ Therefore, despite the majority of osteoarthritis research focusing on the impact on articular cartilage, these existing studies together with our novel data suggest that bone-related changes in shape should be investigated further, especially in the early stages of disease and when considering disease predisposition. It is however important to note that in the current study, we examined hip shape at ages 60–64 years when the cohort are arguably still relatively young and osteoarthritis prevalence was low, and it would therefore be of interest to further examine these potential associations in this cohort at a later date as they continue to age.

In preclinical models, loading of the hip leads to altered joint shape.^28^ The most obvious mechanism by which hip shape may predict osteoarthritis predisposition may also be biomechanical. Indeed, differences in the shape of the hip may influence subsequent joint loading and the forces generated. These in turn likely influence articular cartilage thickness and proteoglycan content.^29^ Further, it is interesting to note that variations in hip shape are also associated with knee osteoarthritis, potentially due to alterations in kinetic chain biomechanics, although a mechanism has yet to be elucidated.^27 30^ Therefore, alternatively, there may be other genetic and molecular mechanisms which may contribute to this relationship. Several known osteoarthritis susceptibility single nucleotide polymorphisms (SNPs) have been associated with hip shape in the ALSPAC, which were within 200 kb of genes involved in endochondral bone formation.^11 12^ Similarly, hip shape in the Genetics, Osteoarthritis and Progression Study was associated with the rs12885300 SNP of *DIO2*, an osteoarthritis susceptibility gene involved in prenatal and postnatal joint development and ultimately endochondral ossification.^31^ A necessary line of research is therefore to evaluate the interaction of developmental processes in determining joint health in later life.

The SITAR growth curve model offers a unique opportunity to effectively examine pubertal growth based on three parameters of size, tempo and velocity in human cohorts.^18 19^ We have previously shown that in the MRC NSHD, there was limited evidence to suggest that height in childhood, as modelled using the SITAR parameters, is associated with odds of knee osteoarthritis in midlife.^13^ Our results presented here similarly suggest that while associations between the SITAR parameters and hip shape are modest, individuals who are taller, have later pubertal timing (later height tempo) and/or faster growth velocity have features including a wider and flatter femoral head and neck, and increased external rotation. Sex‐specific variation was only identified for associations with HM2 (SITAR height size and velocity, and conditional height gain at age 4–7 years), HM4 and HM7 (conditional height gain at age 7–15 years). In all cases, associations were stronger in males, consistent with the studies examining HM by Frysz *et al* 14 and Ireland *et al*,^15^ and with previous studies of physical activity and bone.^32^ These sex differences could reflect a differential response to loading or potential hormonal effects.

Analyses of ALSPAC data have found height tempo to be associated with many of the top hip shape modes at age 14 years, with associations stronger in male than female participants.^14^ However, the authors found little evidence of relationships between height tempo and femoral shape measured at age 18 years.^14^ This is consistent with our studies as here, we found the most consistent pattern of associations with hip shape in later life in the adolescent period of 7–15 years. It has been suggested by Bass *et al* that during periods of fast growth, specific bone regions may be more responsive to genetic and/or environmental stimuli.^33^ Consistent with this, we have previously shown that an early age at onset of walking in infancy is associated with hip shape features which represent an osteoarthritis-like phenotype and suggest that this may relate directly to skeletal loading during a period where skeletal growth is more rapid than at any other time point.^15^ Similarly, there are several studies suggesting that high levels of physical activity during another key period of fast growth, that is, puberty, may have undesirable effects on the hip shape including cam morphology.^10^ Further, these studies suggest that these cam deformities are persistent beyond growth plate closure.^34 35^ Further studies are required to fully define the association and underlying mechanisms between growth during these periods and hip shape deformities.

### Strengths and limitations

In this study, while a number of associations were found, there is a need for replication of these findings in other cohorts as we acknowledge that some may be false positives. However, there are challenges with this as there are a lack of datasets with the relevant data. In this cohort at 60–64 years, the incidence of osteoarthritis was low with most participants having a Kellgren-Lawrence grading of 0.^36^ While this may allow for associations to be examined prior to osteoarthritis pathology, it may also represent selection bias with individuals attending a clinic for DXA assessment more likely to be in better health than those who were visited at home.^37^ A potential limitation of this research is the age at which height was measured, as there is a difference in timing of puberty between males and females. The period of 7–15 years captures the pubertal growth spurt for more females than males, who have later puberty on average. We therefore also included the SITAR parameters in our analyses which model the growth curves of individuals and *height velocity* represents the speed of the pubertal growth spurt and *height tempo* the timing of puberty. Conclusions drawn have therefore considered both these approaches. Another potential limitation is that cross-comparison of individual HMs between studies is difficult due to variation in the different models applied and the sensitivity of the models to the data they draw on. Therefore, while correlations between our results found here and others can be made as the variation in each HM score can be related to specific features of variation, any conclusive comparisons are difficult. Finally, as with all observational studies, causality cannot be assumed; there is a possibility of residual confounding and results found may be due to chance given multiple testing. The major strength of this study is the availability of multiple height measurements prospectively assessed during childhood and adolescence, as well as hip shape SSM modes in a relatively large sample of people at age 60–64 years. Together, this provided a unique opportunity to study the relationships between longitudinal growth and hip shape, through two complementary methods: using (1) the SITAR growth curve model to summarise variables of height size, tempo and velocity; and (2) a conditional change approach to determine sensitive periods of growth which are associated with joint shape.

## Conclusions

In this relatively large population-based cohort study, we have found some evidence to suggest that growth patterns in childhood and adolescence may be associated with modest variations in hip shape at ages 60–64 years, which are consistent with features seen in osteoarthritis. This work contributes to a growing body of work examining how life course height may predict musculoskeletal health in ageing. Further studies are required to fully understand how sensitive periods of growth during childhood and adolescence may relate to joint shape and ultimately health in ageing.

10.1136/rmdopen-2023-003816.supp2 Supplementary data

## Data Availability

Data may be obtained from a third party and are not publicly available. Data used in this publication are available to bona fide researchers upon request to the NSHD Data Sharing Committee via a standard application procedure. Further details can be found at http://www.nshd.mrc.ac.uk/data; doi: 10.5522/NSHD/Q101 and 10.5522/NSHD/S102A.
