# Supplementary material for: Associations between life course longitudinal growth and hip shapes at ages 60–64 years: evidence from the MRC National Survey of Health and Development
Source: RMD Open. 2024 Apr 10;10(2):e003816. doi: 10.1136/rmdopen-2023-003816 (PMC11015210; doi:10.1136/rmdopen-2023-003816)

Supplementary data: Associations between life course longitudinal growth and hip shapes at age 60 to 64: evidence from the 1946 British birth cohort study

Katherine A. Staines., Fiona Saunders., Alex Ireland., Richard Aspden., Jenny Gregory., Rebecca Hardy., Rachel Cooper

Corresponding author: Katherine A. Staines. K.staines@brighton.ac.uk

Table S1. *Unadjusted differences in height (per 5cm) and SITAR parameters (per 1 unit change in height size (cm), and velocity (%)) with hip shape mode, where there was evidence of deviations from linearity when a quadratic term was included in the main model.*

Mode	Height / 5cm at age		RC	95% CI		P
HM8	7	Linear	0.424	-0.096	0.752	0.01
		Quadratic	-0.002	-0.003	-0.000	0.02
HM9	6	Linear	0.371	0.033	0.709	0.03
		Quadratic	-0.002	-0.003	-0.000	0.03
HM9	20	Linear	0.255	0.071	0.438	0.007
		Quadratic	-0.009	-0.001	-0.000	0.005
	**SITAR**					
HM9	Height size	Linear	-0.012	-0.021	-0.003	0.01
		Quadratic	-0.002	-0.003	-0.001	0.005
HM10	Height velocity	Linear	-0.006	-0.011	-0.001	0.02
		Quadratic	0.000	0.000	0.001	0.03

Table S2: *Associations between height (per 5cm) at different ages throughout childhood, adolescence and young adulthood and each hip shape mode. All models were run on the sample with valid data for hip shape modes, height at the specific age and the confounders, adjusted for sex (unless sex interactions were observed). n = 2 years – 1113; 4 years – 1248; 6 years – 1186; 7 years – 1247; 11 years – 1271; 15 years – 1183; 20 years – 1201.*

Mode	Height / 5cm at age	RC	95% CI		P
**HM1**	2	-0.022	-0.083	0.040	0.5
	4	-0.048	-0.104	0.008	0.1
	6	-0.087	-0.143	-0.031	**0.002**
	7	-0.072	-0.124	-0.020	**0.007**
	11	-0.047	-0.087	-0.007	**0.02**
	15	-0.022	-0.059	0.015	0.3
	20	-0.050	-0.095	-0.005	**0.03**
**HM2**	2	0.245	-0.037	0.086	0.4
	4	0.012	-0.045	0.068	0.7
	6 (M)	-0.029	-0.115	0.057	0.5
	6 (F)	0.115	0.041	0.190	**0.003**
	7 (M)	-0.057	-0.135	0.022	0.2
	7 (F)	0.079	0.009	0.149	**0.03**
	11 (M)	-0.065	-0.125	0.005	**0.03**
	11 (F)	0.085	0.032	0.139	**0.002**
	15 (M)	-0.032	-0.078	0.014	0.2
	15 (F)	0.040	-0.024	0.103	0.2
	20 (M)	-0.079	-0.145	-0.013	**0.02**
	20 (F)	0.022	-0.039	0.084	0.5
**HM3**	2	-0.029	-0.089	0.031	0.3
	4	-0.044	-0.099	0.011	0.1
	6	-0.034	-0.088	0.020	0.2
	7	-0.057	-0.107	-0.064	**0.03**
	11	-0.034	-0.074	0.008	0.09
	15	-0.030	-0.066	0.006	0.1
	20	-0.015	-0.058	0.028	0.5
**HM4**	2	-0.042	-0.102	0.019	0.2
	4	-0.023	-0.079	0.032	0.4
	6	-0.045	-0.100	0.009	0.1
	7	-0.061	-0.113	-0.100	**0.02**
	11	-0.061	-0.100	-0.021	**0.003**
	15	-0.074	-0.110	-0.037	**<0.001**
	20	-0.034	-0.077	0.010	0.1
**HM5**	2	0.101	0.039	0.164	**0.001**
	4	0.107	0.050	0.164	**<0.001**
	6	0.108	0.052	0.165	**<0.001**
	7	0.093	0.040	0.146	**0.001**
	11	0.077	0.037	0.118	**<0.001**
	15	0.039	0.002	0.077	**0.04**

	20	0.066	0.021	0.116	**0.004**
**HM6**	2	0.116	0.057	0.176	**<0.001**
	4	0.165	0.111	0.220	**<0.001**
	6	0.148	0.094	0.202	**<0.001**
	7	0.156	0.105	0.207	**<0.001**
	11	0.112	0.072	0.151	**<0.001**
	15	0.110	0.074	0.146	**<0.001**
	20	0.161	0.118	0.205	**<0.001**
**HM7**	2	-0.048	-0.111	0.015	0.1
	4	-0.041	-0.098	0.016	0.2
	6	-0.068	-0.124	-0.012	**0.02**
	7	-0.040	-0.093	0.013	0.1
	11	-0.022	-0.063	0.018	0.3
	15	0.016	-0.021	0.054	0.4
	20	-0.001	-0.045	0.044	1.0
**HM8**	2	0.061	-0.001	0.123	**0.05**
	4	0.025	-0.032	0.081	0.4
	6	0.031	-0.025	0.086	0.3
	7	0.036	-0.016	0.088	0.2
	11	0.043	0.003	0.083	**0.03**
	15	0.026	-0.010	0.062	0.2
	20	0.049	0.004	0.093	**0.03**
**HM9**	2	-0.117	-0.179	-0.055	**<0.001**
	4	-0.071	-0.127	-0.015	**0.01**
	6	-0.081	-0.137	-0.025	**0.004**
	7	-0.102	-0.155	-0.049	**<0.001**
	11	-0.083	-0.123	-0.042	**<0.001**
	15	-0.064	-0.101	-0.026	**0.001**
	20	-0.043	-0.088	0.002	0.06
**HM10**	2	-0.034	-0.093	0.023	0.2
	4	-0.037	-0.090	0.017	0.2
	6	-0.009	-0.062	0.044	0.7
	7	-0.045	-0.095	0.005	0.8
	11	-0.037	-0.075	0.001	0.06
	15	-0.035	-0.067	0.000	**0.05**
	20	-0.065	-0.108	-0.023	**0.002**

Table S3: *Associations between each parameter of the SITAR model of growth curve analysis (per 1 unit change in height size (cm), tempo (years) and velocity (%)) and HM1-10. All models were run on the sample with valid data for each hip shape mode, each SITAR variable and the confounders. Data presented are from linear regression models adjusted for Model 1: sex (unless sex interactions were observed); Model 2: birth weight, sporting ability and Father's occupational class in childhood. n=1380 (667 male and 713 female).*

		**Model 1**				**Model 2**			
**Mode**	**SITAR**	**RC**	**95% CI**		**P**	**RC**	**95% CI**		**P**
**HM1**	Size	-0.010	-0.019	-0.001	**0.03**	-0.012	-0.021	-0.002	**0.015**
	Tempo	0.003	-0.005	0.010	0.5	0.002	-0.006	0.010	0.6
	Velocity	-0.003	-0.008	0.002	0.2	-0.004	-0.009	0.001	0.2
**HM2**	Size (M)	-0.020	-0.034	-0.007	**0.03**	-0.021	-0.035	-0.007	**0.003**
	Size (F)	0.004	-0.008	0.017	0.5	0.004	-0.009	0.017	0.6
	Tempo	-0.011	-0.019	-0.003	**0.005**	-0.011	-0.019	-0.004	**0.004**
	Velocity (M)	-0.012	-0.020	-0.004	**0.002**	-0.012	-0.020	-0.004	**0.002**
	Velocity (F)	0.002	-0.005	0.009	0.6	0.002	-0.005	0.009	0.6
**HM3**	Size	-0.004	-0.013	0.005	0.4	-0.001	-0.010	0.008	0.8
	Tempo	0.005	-0.003	0.013	0.2	0.005	-0.003	0.012	0.2
	Velocity	0.001	-0.004	0.006	0.8	0.001	-0.004	0.006	0.6
**HM4**	Size	-0.011	-0.019	-0.002	**0.02**	-0.009	-0.019	-0.000	**0.05**
	Tempo	0.007	-0.001	0.015	0.07	0.007	-0.001	0.015	0.07
	Velocity	-0.006	-0.011	-0.001	**0.02**	-0.006	-0.011	-0.007	**0.03**
**HM5**	Size	0.015	0.005	0.024	**0.002**	0.014	0.004	0.023	**0.006**
	Tempo	-0.003	-0.011	0.005	0.5	-0.002	-0.010	0.006	0.6
	Velocity	0.002	-0.004	0.007	0.5	0.001	-0.004	0.007	0.6
**HM6**	Size	0.038	0.029	0.046	**<0.001**	0.039	0.030	0.048	**<0.001**
	Tempo	0.003	-0.005	0.011	0.5	0.003	-0.005	0.010	0.5
	Velocity	0.015	0.010	0.020	**<0.001**	0.015	0.010	0.020	**<0.001**
**HM7**	Size	0.000	-0.009	0.009	1.0	0.001	-0.010	0.010	0.9
	Tempo	0.002	-0.006	0.001	0.6	0.002	-0.006	0.010	0.6
	Velocity	0.005	-0.000	0.101	0.07	0.005	-0.000	0.103	**0.06**
**HM8**	Size	0.011	0.002	0.020	**0.02**	0.011	0.002	0.021	**0.02**
	Tempo	0.002	-0.005	0.101	0.6	0.002	-0.005	0.010	0.5
	Velocity	0.003	-0.003	0.008	0.3	0.003	-0.003	0.008	0.3
**HM9**	Size	-0.013	-0.022	-0.004	**0.005**	-0.014	0.023	-0.004	**0.005**
	Tempo	0.010	0.002	0.018	**0.01**	0.010	0.003	0.018	**0.01**
	Velocity	-0.004	-0.009	0.001	0.1	-0.004	-0.009	0.001	0.2
**HM10**	Size	-0.013	-0.022	-0.004	**0.003**	-0.013	-0.022	-0.004	**0.005**
	Tempo	-0.002	-0.009	0.006	0.7	-0.002	-0.009	0.006	0.6
	Velocity	-0.006	-0.011	-0.001	**0.03**	-0.006	-0.010	-0.001	**0.03**

Table S4: *Associations of conditional height gain (per standard deviation) during different periods of growth (early childhood: 2–4 years; childhood to adolescence: 7–15 years; adolescence to young adulthood: 15–20 years) with each hip shape mode. All models were run on the sample with complete data for each hip shape mode, height at each age, and the confounders. Data presented are from linear regression models adjusted for Model 1: sex (unless sex interactions were observed); Model 2: birth weight, sporting ability and Father's occupational class in childhood. n = 648 (319 male and 329 female).*

		Model 1				Model 2			
Mode	Conditional change (years)	RC	95% CI		P	RC	95% CI		P
**HM1**	2 – 4	-0.001	-0.078	0.076	1.0	-0.003	-0.080	0.074	0.9
	4 – 7	-0.049	-0.127	0.028	0.2	-0.052	-0.128	0.024	0.2
	7 – 15	0.014	-0.063	0.091	0.7	-0.008	-0.068	0.084	0.8
	15 – 20	-0.017	-0.093	0.058	0.7	0.006	-0.081	0.069	0.9
**HM2**	2 – 4	-0.011	-0.087	0.065	0.8	-0.012	-0.089	0.067	0.8
	4 – 7 (M)	-0.019	-0.127	0.089	0.7	-0.021	-0.11	0.088	0.7
	4 – 7 (F)	0.071	-0.039	0.181	0.2	-0.047	-0.124	0.029	0.2
	7 – 15	-0.046	-0.122	0.030	0.2	-0.041	-0.102	0.021	0.2
	15 – 20	-0.099	-0.174	-0.024	**0.01**	-0.097	-0.172	-0.022	**0.01**
**HM3**	2 – 4	0.005	-0.069	0.078	0.9	-0.006	-0.069	0.080	0.9
	4 – 7	-0.040	-0.113	0.034	0.3	-0.044	-0.118	0.030	0.2
	7 – 15	-0.012	-0.085	0.061	0.7	0.004	-0.078	0.069	0.9
	15 – 20	-0.009	-0.082	0.063	0.8	-0.005	-0.078	0.067	0.9
**HM4**	2 – 4	-0.016	-0.091	0.059	0.7	0.002	-0.075	0.079	1.0
	4 – 7	-0.078	-0.154	-0.02	**0.04**	-0.071	-0.147	0.05	0.1
	7 – 15 (M)	-0.098	-0.214	0.018	0.1	-0.095	-0.212	0.022	0.1
	7 – 15 (F)	-0.011	-0.107	0.084	0.8	-0.053	-0.103	0.092	0.9
	15 – 20	-0.001	-0.076	0.073	1.0	0.002	-0.073	0.077	1.0
**HM5**	2 – 4	0.090	0.014	0.167	**0.02**	0.080	0.002	0.159	**0.04**
	4 – 7	0.045	-0.033	0.123	0.3	0.041	-0.037	0.119	0.3
	7 – 15	-0.057	-0.134	0.020	0.1	-0.061	-0.138	0.017	0.1
	15 – 20	0.052	-0.024	0.129	0.2	0.043	-0.033	0.119	0.3
**HM6**	2 – 4	0.170	0.096	0.243	**0.0**	0.165	0.090	0.240	**0.0**
	4 – 7	0.048	-0.027	0.123	0.2	0.042	-0.033	0.118	0.3
	7 – 15	0.064	-0.101	0.138	0.1	0.063	-0.012	0.138	0.1
	15 – 20	0.800	0.007	0.153	**0.03**	0.078	0.005	0.152	**0.04**
**HM7**	2 – 4	-0.041	-0.118	0.036	0.3	-0.027	-0.106	0.051	0.5
	4 – 7	-0.039	-0.117	0.039	0.3	-0.032	-0.110	0.046	0.4
	7 – 15 (M)	0.171	0.067	0.276	**0.001**	0.168	0.062	0.274	**0.002**
	7 – 15 (F)	0.015	-0.097	0.126	0.8	0.022	-0.092	0.135	0.7
	15 – 20	-0.070	-0.083	0.069	0.9	0.000	-0.076	0.076	1.0
**HM8**	2 – 4	0.024	-0.051	0.100	0.5	0.037	-0.040	0.114	0.4
	4 – 7	0.013	-0.063	0.089	0.7	0.016	-0.060	0.092	0.7
	7 – 15	-0.017	-0.092	0.058	0.7	-0.011	-0.087	0.065	0.8
	15 – 20	0.062	-0.012	0.136	0.1	0.066	-0.009	0.140	0.08
**HM9**	2 – 4	-0.043	-0.120	0.034	0.3	-0.030	-0.109	0.049	0.5
	4 – 7	-0.030	-0.108	0.048	0.5	-0.025	-0.103	0.053	0.5
	7 – 15	-0.018	-0.095	0.059	0.6	-0.009	-0.087	0.068	0.8

	15 – 20	0.066	-0.010	0.142	0.09	0.068	-0.008	0.145	0.08
**HM10**	2 – 4	-0.027	-0.096	0.042	0.4	-0.021	-0.092	0.049	0.6
	4 – 7	-0.032	-0.101	0.038	0.4	-0.029	-0.099	0.041	0.4
	7 – 15	-0.043	-0.112	0.026	0.2	-0.041	-0.111	0.029	0.2
	15 – 20	-0.053	-0.121	0.016	0.1	-0.052	-0.120	0.017	0.1

Table S5: *Shape variations described by the associations with each of the HM and the SITAR variables and conditional height periods.*

**Greater SITAR height size**	
HM1 negative	More compact femoral head<br>Larger neck-shaft angle
HM2 negative (male only)	Longer femoral neck<br>Increased external rotation<br>Loss of femoral head curvature
HM9 negative	Wider femoral neck<br>Increasing osteophytes<br>More compact femoral head
HM10 negative	Flatter femoral neck curvature<br>Medial enlargement of femoral head<br>Narrower femoral shaft
HM6 positive	Some evidence of external rotation from positive to negative scores
HM8 positive	Slight medial migration of femoral head<br>Slightly larger lesser trochanter
**Later SITAR height tempo**	
HM2 negative	Longer femoral neck<br>Increased external rotation<br>Loss of femoral head curvature
**Greater SITAR height velocity**	
HM2 negative (male only)	Longer femoral neck<br>Increased external rotation<br>Loss of femoral head curvature
HM10 negative	Flatter femoral neck curvature<br>Medial enlargement of femoral head<br>Narrower femoral shaft
HM6 positive	Some evidence of external rotation from positive to negative scores
HM7 positive	Wider, flatter femoral head<br>Shorter femoral neck<br>Slight external rotation
**Conditional change 2 – 4 years**	
HM6 positive	Some evidence of external rotation from positive to negative scores
**Conditional change 7 – 15 years**	
HM2 negative	Longer femoral neck<br>Increased external rotation<br>Loss of femoral head curvature
HM5 negative	Possible external rotation (more of the lesser trochanter visible)
HM6 positive	Some evidence of external rotation from positive to negative scores
HM7 positive (males only)	Wider, flatter femoral head<br>Shorter femoral neck<br>Slight external rotation
**Conditional change 15 – 20 years**	
HM2 negative	Longer femoral neck<br>Increased external rotation<br>Loss of femoral head curvature

Figure S1: *Flow chart of cohort numbers in this analysis*

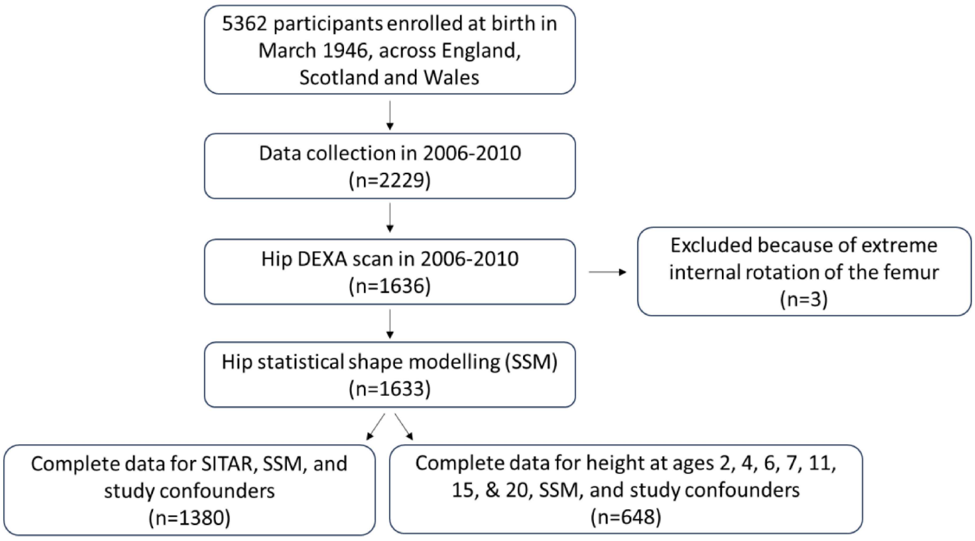

Figure S2: *Scatter plots of weighted regression of height (per 5cm) and SITAR parameters with hip shape mode where deviations from linearity were observed.,*

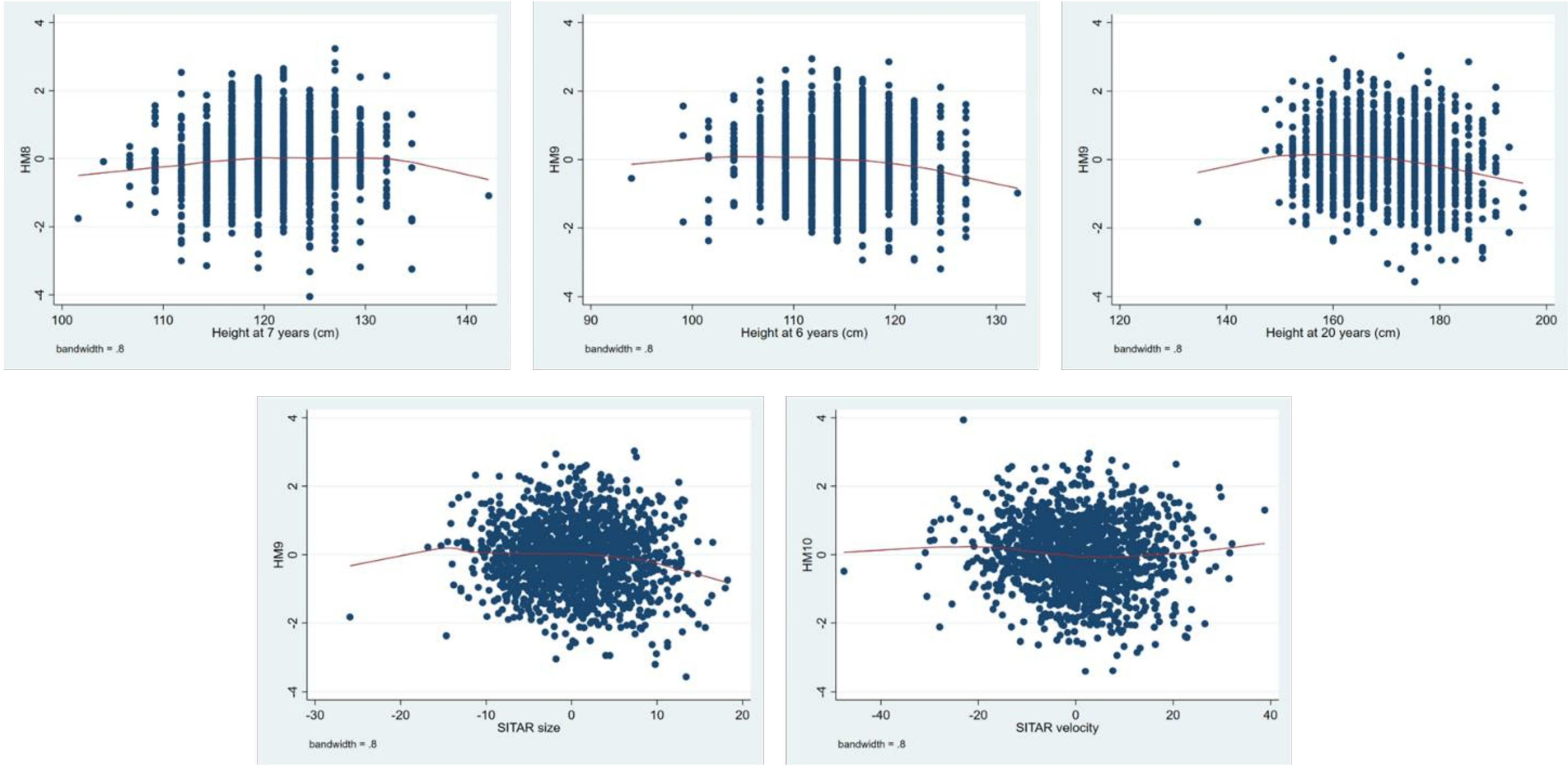

Figure S3: *Scatter plots of weighted regression of height (per 5cm) with hip shape mode where associations were observed in Table S2.*

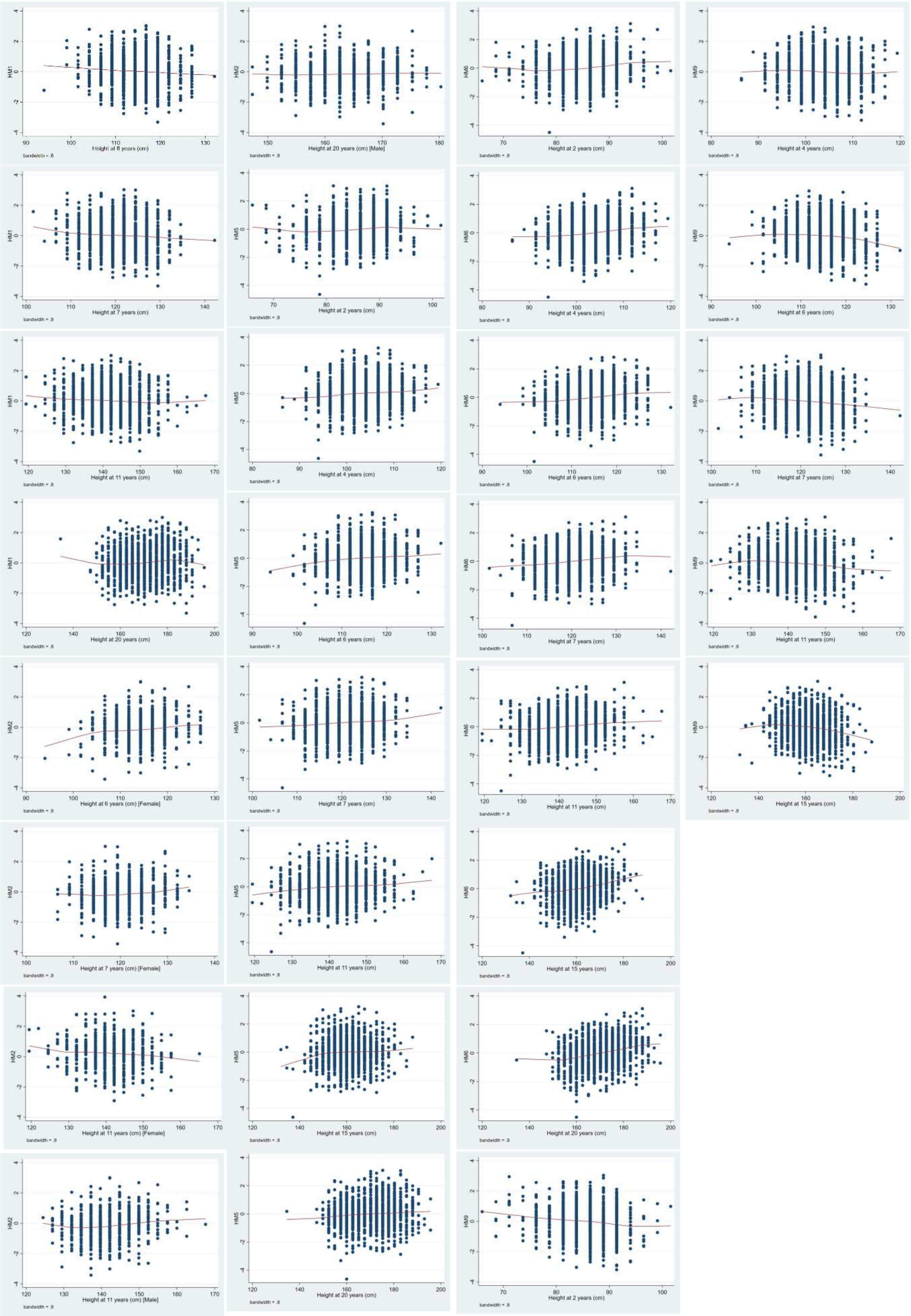

Figure S4: *Scatter plots of weighted regression of SITAR parameters with hip shape mode where associations were observed in Table 2.*

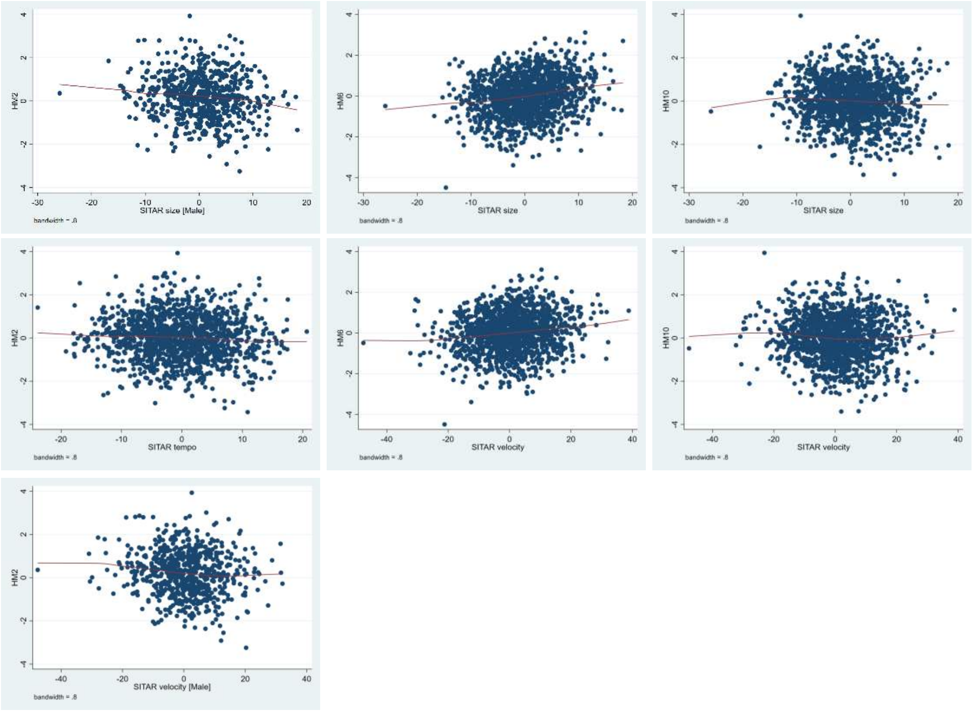

Supplement: Supplementary data [file rmdopen-2023-003816supp001.pdf]
